# Large bilateral tubercular pyosalpinx in a young woman with genitourinary malformation: a case report

**DOI:** 10.1186/1752-1947-8-176

**Published:** 2014-06-03

**Authors:** Jacqueline Gascón, Pedro Acién

**Affiliations:** 1 Department of Obstetrics and Gynecology, San Juan University Hospital, Alicante, Spain; 2 Department/Division of Gynecology, School of Medicine, Miguel Hernandez University, Campus of San Juan, 03550 Alicante, Spain

**Keywords:** Genital tuberculosis, Hysterosalpingography, Pelvic kidney, Pyosalpinx, Infertility, Uterine malformation

## Abstract

**Introduction:**

Tuberculosis is a chronic infectious disease, and the morbidity associated with it has major health implications. When tuberculosis affects the genital organs of young females, it has the devastating effect of causing irreversible damage to their fallopian tubes, resulting in a possible tubercular pyosalpinx and infertility. However, the disease often remains silent or presents with very few specific symptoms. In adolescents and young women, tuberculosis can also present with hypogastric recurrent symptoms and affectation of the general state, but because in our country genital tuberculosis is uncommon, its diagnosis is unlikely.

**Case presentation:**

We describe the case of an 18-year-old Spanish woman who had been sexually active for 1 year, nulliparous, who presented with hypogastric discomfort and repeated urinary symptoms complicated with pelvic inflammatory disease after a hysterosalpingography. Genital tuberculosis was not suspected. The echographical findings and tumor markers mimicked those of ovarian tumors, and she was also a carrier of a genitourinary malformation (pelvic kidney and septate uterus). A laparotomy was performed and revealed large pelvic abscesses. On her right adnexum, the large pyosalpinx was free (floating pyosalpinx). Drainage, adhesiolysis and bilateral salpingectomy were performed, and cultures were taken. Histopathological study showed bilateral granulomatous abscessificated salpingitis with suspicion of genital tuberculosis, and cultures were positive for *Mycobacterium tuberculosis*. She followed a tuberculostatic treatment for 6 months. Eight years later, she presents with normal menstruations and is waiting for an *in vitro* fertilization cycle.

**Conclusions:**

No other reported case showing similar association of genital tuberculosis and genitourinary malformation was found. The associated genitourinary malformation in this case probably has no relation but it could contribute to diagnosis delay and/or to reactivate the pathology. The hysterosalpingographical findings and the observation of a floating pyosalpinx must alert the clinician to genital tuberculosis, but the diagnosis is suggested by the histopathological studies and confirmed by cultures. In this case study, the necessity of considering the risk of pelvic inflammatory disease reactivation after hysterosalpingography, of suspecting the diagnosis of genital tuberculosis and of establishing the differential diagnosis with ovarian tumors in the presence of large pyosalpinges is highlighted.

## Introduction

Tuberculosis (TB) is a chronic infectious disease and the morbidity associated with it has major health implications. The disease has a worldwide distribution, but its incidence is higher in developing countries. In our environment, an increase in the incidence of TB has occurred in recent years, which is essentially attributed to human immunodeficiency virus (HIV) infection, immigration from zones with a high prevalence of acquired immunodeficiency syndrome (AIDS), drug addiction and low economic social status, occupational exposure and lack of immunization with Bacille de Calmette et Guérin (BCG). Furthermore, TB is considered a criterion for the development of AIDS [1].

When TB affects the genital organs of young females, it has the devastating effect of causing irreversible damage to the fallopian tubes, resulting in infertility that is difficult to cure both by medical and surgical methods [2]. However, the disease often remains silent or may present itself with very few specific symptoms. As a result, the prevalence of genital tuberculosis (GTB) is most likely underestimated and varies from less than 1% among infertile women in the United States of America (USA) and other developed countries to nearly 18% in African and Indian countries [2,3].

GTB usually occurs at the age of menarche and adolescence or after full sexual development but goes unnoticed and is usually diagnosed years after [1]. Infertility is the most common presentation of the GTB and it varies from 10 to 85% [4]. This pathology not only causes tubal obstruction but also impairs implantation due to endometrial involvement and to ovulatory failure from ovarian involvement. Synechiae of the uterine cavity can also be the cause of infertility, and TB peritonitis has also been seen in combination with GTB in 45 to 50% of patients with GTB [5]. Even latent GTB may be a cause of repeated *in vitro* fertilization (IVF) failure if the disease is not diagnosed and treated prior to conducting IVF [6].

The clinical manifestations of GTB include moderate and chronic hypogastric pain along with possible menstrual disorders, or it can present with more general symptoms. In adolescents and young women, GTB can also present with affectation of the general condition, such as fever or low-grade fever, anorexia and weight loss, ascites, adnexal masses, swelling of the peritoneum, diarrhea, constipation or dyspepsia, and, frequently, an increased serum cancer antigen (CA)-125 level. In fact, peritoneal TB can mimic advanced ovarian cancer because of the similarities in clinical signs and symptoms, including the elevation of serum CA-125 levels, of these two diseases [7]. However, urinary symptoms have rarely been noted as a clinical presentation of GTB.

As indicated, in developed countries, GTB can be seen in young women who present with infertility. In these patients, the hysterosalpingography (HSG) findings can be unilateral/bilateral tubal block (58%), unilateral or bilateral hydrosalpinx (19%), dilated tubes (12%), or others [8], and these findings can suggest the diagnosis of GTB. However, the diagnosis of GTB is much more difficult if the clinician does not suspect GTB. Therefore, this disease is often diagnosed incidentally. Other incidental findings in HSG have included an arcuate and septate uterus with bilateral tuberculous salpingitis [8,9]. However, as far as we know, an association of GTB with congenital combined genitourinary malformations has not been described previously.

Here we present the case of a young woman with severe GTB presenting with hypogastric discomfort and repeated urinary symptoms, complicated with pelvic inflammatory disease (PID) after an HSG study. The echographical findings and tumor markers mimicked those of ovarian tumors (borderline), and the woman was also a carrier of a genitourinary malformation (pelvic kidney and septate uterus).

## Case presentation

**Figure 1:**
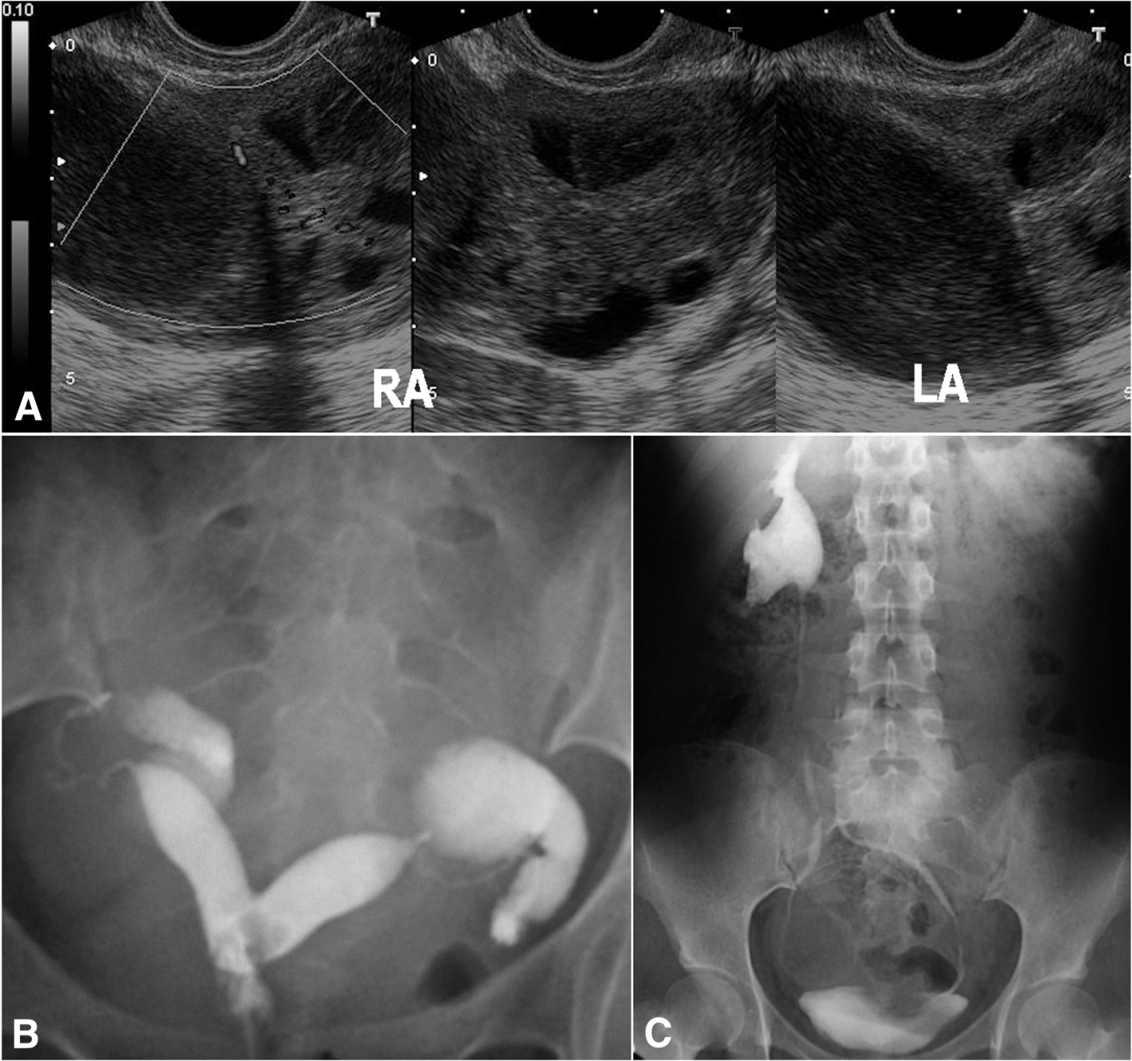
**Diagnostic imaging tests. A**. Transvaginal ultrasound findings; RA, right adnexum; LA, left adnexum. **B**. Hysterosalpingographic image showing bicornuate or subseptate uterus and bilateral hydrosalpinx. **C**. Intravenous pyelography showing the left pelvic kidney at the height of the promontory.

**Figure 2:**
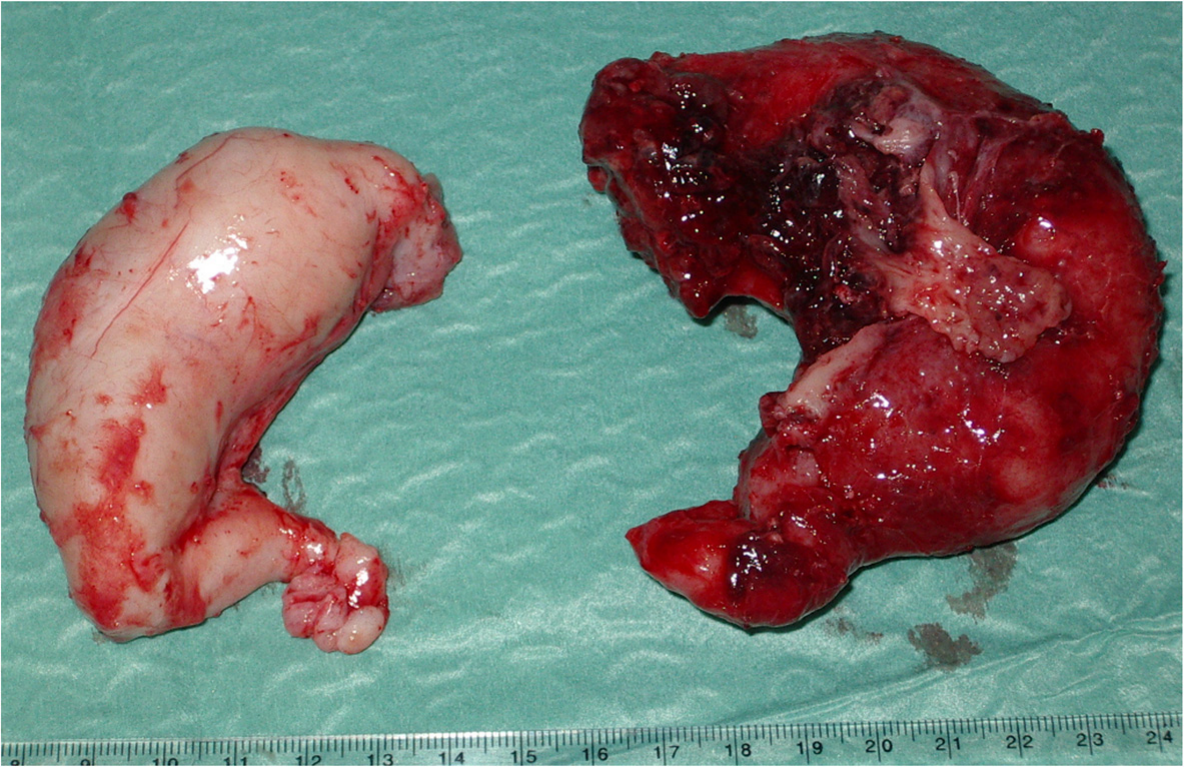
Both fallopian tubes showing large tubercular pyosalpinges.

An 18-year-old Spanish woman, nulliparous, who had been sexually active for 1 year, was transferred from the urology department to our out-patient surgery department in May 2005. At the urology department, she had been seen a year prior due to repeated urinary infections, perhaps related to the presence of an ectopic left pelvic kidney. In June 2004, as a consequence of persistent discomfort in her iliac left fossae, she underwent an intravenous pyelography (IVP), which showed her left pelvic kidney (at the level of S1). In December 2004 and January 2005, she was once again admitted to the emergency department for hypogastric pain and a urinary infection; she was hospitalized and diagnosed with pyelonephritis. Using an ultrasound scan and computed tomography, she was also diagnosed with a bilateral hydrosalpinx and genitourinary malformation: a pelvic kidney and a suggested bicornuate uterus. Under antibiotic treatment (ciprofloxacin and tobramycin), her symptoms decreased, and she returned home with only occasional pain in her hypogastric region and iliac fossae.The gynecological examination was nearly normal. Her uterus was in retroversion, but with transvaginal ultrasound (TVU) it was noticed to have a bicornuate appearance. Her ovaries were apparently normal, but there were bilateral tumors located anteuterine and they were elongated, of 6 to 7cm, suggesting endometriomas or mucinous tumors by their appearance. Cervicovaginal cultures were taken, and analysis, a new IVP and an HSG study were requested. The cultures were negative and the general analyses were normal, except for blood sedimentation rate (BSR)=36mm/hour and serum CA-125=98U/mL. The IVP showed a normal right kidney but a left pelvic kidney, and she had to undergo antibiotic treatment comprising Vibramycin® (doxycycline) and anti-inflammatory agents; thereafter an HSG was performed. This HSG showed that her uterus had a bicornuate appearance and bilateral hydrosalpinx (see Figure 1), but after this test was performed she was admitted to the hospital for the possible reactivation of PID. Once again, the cultures were negative for syphilis and HIV but her BSR was 66 and she had a low-grade fever (37° to 37.5°C). She was given antibiotic treatment comprising clindamycin and Vibramycin® (doxycycline); the repeated TVU was similar to previous ones, showing bilateral solid-cystic tumors, such as abscesses, endometriomas or mucinous tumors, with increased Doppler vascularization, but with resistance index=0.7 (November 2005). After clinical improvement, a programmed surgical intervention was proposed and, in the meantime, she was discharged from the hospital. One month later, her BSR was 44 and her serum CA-125=424U/mL. At the time, the elective laparotomy was performed (December 2005) and revealed large pelvic abscesses located anteuterus, especially on the left side, with converged tubes, the bladder, the sigmoid colon and a large left pyosalpinx. On the right adnexum, the large pyosalpinx was free (floating pyosalpinx). Her ovaries showed some adhesions but seemed normal; her uterine fundus was also normal indicating a septate uterus. The rest of her abdominal cavity and gastrointestinal tract seemed normal. Drainage, adhesiolysis and bilateral salpingectomy (see Figure 2) were performed and cultures were taken. We touched her left kidney at the height of the promontory. The postoperative course was normal.

**Figure 3:**
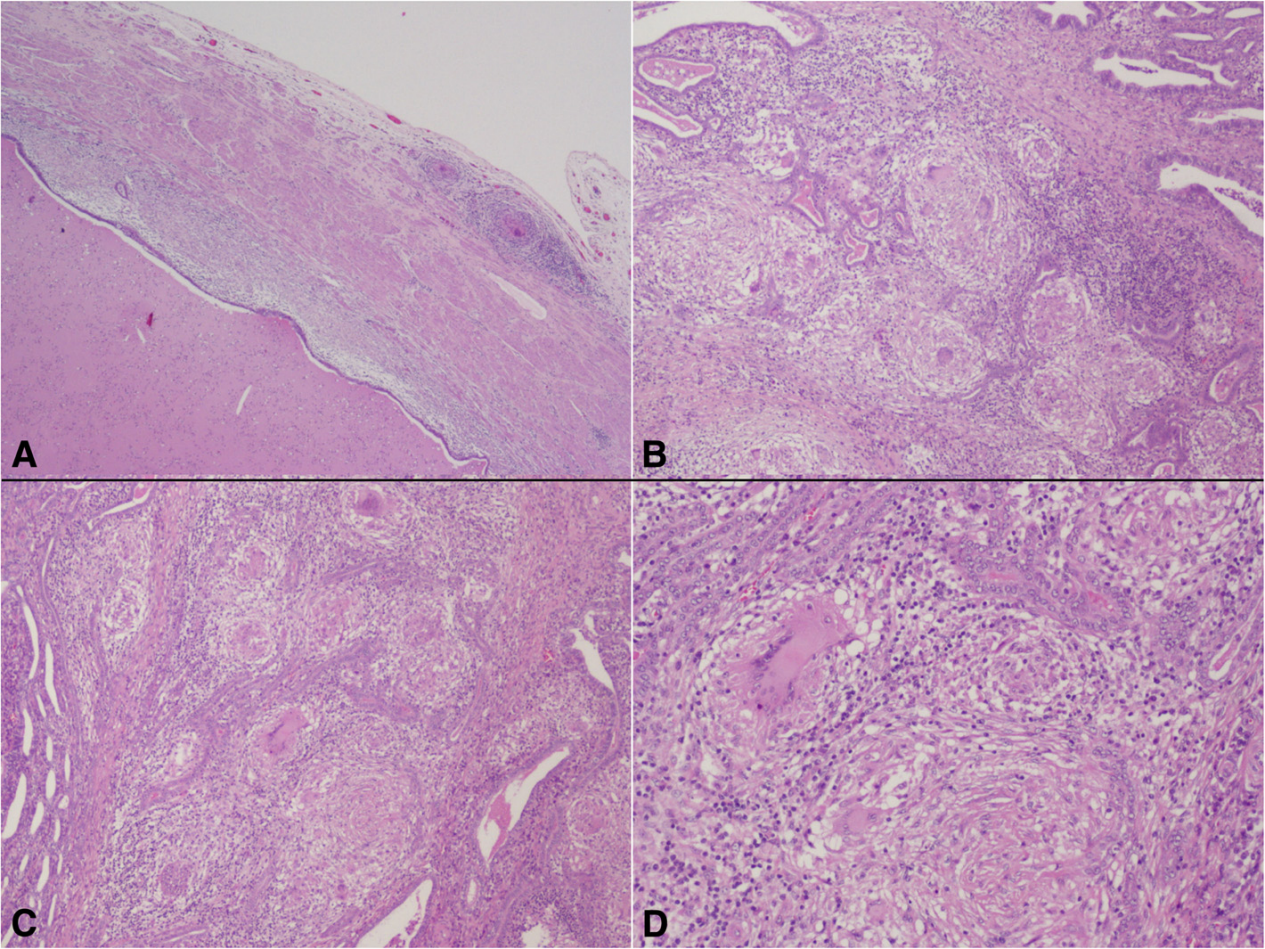
**Histopathological findings. A**. Pyosalpinx. Section of the tubal wall and lumen, hematoxylin and eosin 40×. **B**. and **C**. Tubal wall showing tubercular granulomas, hematoxylin and eosin 100×. **D**. Detail of a tubercular granuloma, hematoxylin and eosin 200×.

The cultures of the abscess samples taken during the laparotomy initially showed the following: Gram stain, negative; Ziehl–Neelsen stain, negative for alcohol-acid resistant bacilli; and culture for aerobic and anaerobic germs, negative. The samples continued to be cultured and, after several days, were found to be positive for *Mycobacterium tuberculosis*, which was in agreement with the histopathological result. The culture in liquid medium gave a similar positive result and the samples that were sent to a reference center were found to be sensitive to anti-TB drugs (streptomycin, rifampicin, isoniazid, ethambutol, and pyrazinamide). The histopathological study showed bilateral granulomatous abscessificated salpingitis with suspicion of GTB (see Figure 3).

Then, after the TB infection was confirmed, she was referred to the Infectious Diseases Unit of our hospital where she followed a tuberculostatic treatment with Rimcure (rifampicin, isoniazid and pyrazinamide) and afterwards Rifinah® (rifampicin and isoniazid) for 6 months. The sputum culture obtained before the beginning of the treatment was negative for *Mycobacterium tuberculosis*, and the Ziehl–Neelsen stain and the urine cultures (in Löwenstein–Jensen medium and liquid medium) performed in April 2006 were also negative. She continued to have normal menstruation and after having finished the 6 months of treatment in October 2006 she did not return for further follow-up.

Four years later (October 2010), the now 23-year-old patient was again sent to our gynecology out-patient department due to discomfort in her left iliac fossae, dysmenorrhea and the suspicion of an ovarian cyst. The results of the gynecological examination, the TVU and all analyses practiced were normal. She was attempting to become pregnant and in February 2011 she requested assisted reproduction techniques; she was remitted to the corresponding unit for IVF. Afterwards, she did have a pregnancy but it ended in abortion. The serological analyses for toxoplasmosis, syphilis and HIV-AIDS conducted in February 2012 were negative (rubella positive). Presently, she presents with normal menstruations and is waiting for a new IVF cycle.

## Discussion

As indicated above, GTB often remains silent or may present with very few specific symptoms. Even so, its diagnosis is more difficult and is delayed if clinicians do not consider it a possibility. This is especially true because GTB is a pathology that mimics other diseases. GTB can be confused with different gynecological pathologies, such as PID, ovarian torsion, ectopic pregnancy, bleeding cysts, tubo-ovarian masses or infected ovarian cysts, or with non-gynecological pathologies, such as acute appendicitis, other granulomatous processes, actinomycosis, or urinary tract infections [10]. However, urinary symptoms have rarely been noted as a clinical presentation of GTB.

The case report described here presents several interesting facts that should be considered and discussed: 1) the lack of TB predisposing factors, the uncommon form of presentation, and the difficulty of its diagnosis and of differential diagnosis led to GTB not being considered; 2) the bilateral large pyosalpinx findings, most probably by superimposed PID, and their confusion with ovarian tumors; and 3) the association of GTB with a genitourinary malformation (ectopic pelvic kidney and septate uterus), which most probably had no relation to the TB infection, but contributed, nonetheless, to the diagnostic confusion and the delay in obtaining a correct diagnosis.

Regarding the first point, the patient did not have a personal nor family history that could suggest a TB infection. She also did not have the known risk factors for GTB (AIDS, immigration or drug addiction), as we noted above, in our country GTB is uncommon; despite the HSG findings, the clinicians did not consider a diagnosis of GTB. As was shown by Ghosh *et al*. [5], “though finer diagnostic tools of detection of TB are increasingly available in the form of bacterial cultures and polymerase chain reaction (PCR) based diagnostic suspicion by clinicians remains the main tool for diagnosis of the condition”. It has previously been stated that the presentation of TB in women makes its diagnosis difficult because many women are asymptomatic [10]. In the case studied here, the TB symptoms were also limited to hypogastric discomfort and repeated urinary infections along with occasional exacerbations (pyelonephritis) attributed to the presence of one ectopic pelvic left kidney. Primary infection of the genital tract by TB is extremely unusual and it is almost always secondary to other infections [10]. Almost always, the infection is produced by inhalation of pulmonary infectious TB. This primary infection, in most cases, is controlled by the immune response; the damage caused by the infection tends to be fibrosis and calcification. However, in the 2 or 3 weeks that it takes for the specific T-cell-mediated response to develop, asymptomatic hematogenous spread of the bacilli can occur. The bacilli preferentially spread to places with major partial pressure of oxygen and confluent terminal vascularization, including lung vertices and the female genital tract, especially the fallopian tube, where they may remain in a latent state for a long time.

Postprimary or secondary TB occurs due to the reactivation of this latent infection or, more rarely, due to re-infection [1]. The TB bacilli can reach the female genital tract through hematogenous routes, which occurs in 90% of cases from a primary infection of the lungs, lymph nodes or skeletal system [10]. Infection of the female genitourinary tract can also occur through the lymphatic system or directly from the gastrointestinal tract, mesenteric nodes or peritoneum or, rarely, by sexual transmission [10]. We do not know if sexual transmission was the route of infection in this case, but the onset of clinical manifestations more or less coincided with the date of the onset of sexual intercourse for this patient.

However, as indicated, GTB can be asymptomatic for years and can originate as an active pulmonary TB or not. Furthermore, because of the few symptoms of GTB [11,12], only 50% of cases are diagnosed without surgery and, most frequently, the diagnosis becomes apparent as a salpingitis that expresses itself by primary infertility. Other times, GTB is initiated with pelvic pain, by menstrual disorders or, less frequently, the presence of fever and the commitment of a general state. In the most advanced cases, there is involvement of the fallopian tubes, uterus and ovaries, and such genital affectation can develop from one salpingo-oophoritis into a pelvic peritonitis [13,14]. In our case, the pelvic pain was not marked, and it was the occasional urinary symptoms that led to the diagnosis of a pelvic ectopic kidney. Subsequently, symptoms of PID presented after the HSG was performed, but we assumed that these symptoms were due to the reactivation of the chronic PID and responsible for the finding of a bilateral hydrosalpinx, seen previously in the ultrasound scan and in a subsequent HSG. Kulshrestha *et al*. [8] showed previously that the HSG findings of 169 women were principal unilateral/bilateral tubal block (58%), or unilateral or bilateral hydrosalpinx (19%) or dilated tubes (12%). However, a superimposed PID seemed to occur in our case. As mentioned before, TB is considered one of the greatest imitators of other diseases, thus making its diagnosis a clinical challenge [1,15]. Other inflammatory purulent processes, such as endometritis and salpingitis, a ruptured ectopic pregnancy or an acute appendicitis, must be ruled out. Other chronic granulomatous diseases, such as parasitosis, coccidiosis or actinomycosis in patients who have used an intrauterine device for a prolonged period of time or have Crohn’s disease, sarcoidosis, brucellosis, or tularemia, can give problems for the differential diagnosis of TB, but only 50% of TB cases have granulomatous lesions [1,10].

Regarding the second point in the discussion, the finding of a large bilateral pyosalpinx and its confusion with ovarian tumors has been mentioned in other publications. In effect, GTB can present with adnexal tumors, ascites and elevation of the serum CA-125<500UI/mL; it is difficult to establish the differential diagnosis from ovarian cancer. The clinical findings are of little utility because both pathologies can present with the same vague symptoms; furthermore, the elevation of serum CA-125 in both diseases makes the differential diagnosis even more difficult, and the ascitic liquid culture is only positive in approximately 30% of cases [1,14]. Several publications have shown that GTB and TB peritonitis mimic ovarian cancers, but the antigen serum levels of CA-125 and high-molecular-weight glycoproteins have been used to monitor the response of the GTB to anti-TB treatment [10].

Finally, with regard to the third point in the discussion, the analysis of the relation of the GTB with genitourinary malformations, we must note that we have not found publications regarding this observation. Some incidental findings in HSG in the paper by Kulshrestha *et al*. [8] included an arcuate uterus in four (2.4%) women and a uterine septum in two (1.2%). In addition, Balasch *et al*. [9] described a twin pregnancy with term delivery after IVF in a patient with diethylstilbestrol (DES) anomalies, septate uterus and bilateral tuberculous salpingitis. In our case, we believe that the urinary abnormality (pelvic kidney) and the septate uterus contributed greatly to the diagnostic confusion. In addition, our diagnostic investigations involving an HSG probably contributed to the reactivation of the pathology of GTB and the acute associated PID.

## Conclusions

GTB is rare in our environment, which is why clinicians do not typically consider GTB a potential diagnosis. Moreover, no other reported case shows a similar association of GTB and genitourinary malformation. The need to remember the risk of PID reactivation to properly diagnose GTB and to distinguish it from ovarian tumors due to large bilateral pyosalpinx and its possible association with a genitourinary malformation must be highlighted. Histopathological study and cultures are the gold standard for a definitive diagnosis.

### Established facts

TB can affect the genital organs of young females causing irreversible damage to the fallopian tubes and therefore infertility.

The disease often remains silent or presents with very few specific symptoms.

The hysterosalpingographical findings and the observation of a floating pyosalpinx must alert the clinician to the possibility of GTB, but the diagnosis is suggested by the histopathological studies and confirmed by cultures.

### Novel insights in this case report

Although GTB is infrequent, it must be suspected in the presence of vague symptoms and reactivation as a PID after a HSG.

The ultrasound findings and tumor markers (CA-125) can mimic ovarian cancers in the presence of tuberculous pyosalpinx.

An associated genitourinary malformation probably has no relation but it contributed to diagnosis delay and/or to reactivate the pathology.

A possible sexual transmission could be suggested in this case.

## Consent

Written informed consent was obtained from the patient for publication of this case report and accompanying images. A copy of the written consent is available for review by the Editor-in-Chief of this journal.

## Abbreviations

AIDS: Acquired immunodeficiency syndrome; BSR: Blood sedimentation rate; CA: Cancer antigen; GTB: Genital tuberculosis; HIV: Human immunodeficiency virus; HSG: Hysterosalpingography; IVF: *In vitro* fertilization; IVP: Intravenous pyelography; PID: Pelvic inflammatory disease; TB: Tuberculosis; TVU: Transvaginal ultrasound.

## Competing interests

The authors declare that they have no competing interests.

## Authors’ contributions

JG extracted the clinical history, followed the patient, made the review and reviewed the manuscript. PA designed the study, made the surgical operation and followed the patient and wrote the manuscript. PA had full access to all of the data in the study and took responsibility for the integrity of the data and the accuracy of the data analysis. Both authors read and approved the final manuscript.
